# Hemostatic and antibacterial calcium–copper zeolite gauze for infected wound healing†

**DOI:** 10.1039/d3ra06070e

**Published:** 2024-01-02

**Authors:** Mingtao Wang, Wenzhao Zhang, Chenchen Wang, Liping Xiao, Lisha Yu, Jie Fan

**Affiliations:** a Key Lab of Applied Chemistry of Zhejiang Province, Department of Chemistry, Zhejiang University Hangzhou 310027 China yulisha_bless@163.com jfan@zju.edu.cn lpxiao@zju.edu.cn; b Key Laboratory of Precision Diagnosis and Treatment for Hepatobiliary and Pancreatic Tumor of Zhejiang Province, The Second Affiliated Hospital, Zhejiang University School of Medicine Hangzhou 310009 China

## Abstract

The design and development of wound dressings with excellent procoagulant and antibacterial activity to achieve high wound healing effectiveness are highly desirable in clinical applications. In this work, we develop a calcium–copper zeolite gauze (CaCu-ZG) by a two-step process involving calcium and copper ion exchange in a zeolite gauze. The CaCu-ZG exhibits remarkable procoagulant and antibacterial abilities, as well as good biocompatibility. Compared with the medical gauze, the blood clotting time of CaCu-ZG significantly decreases and the antibacterial activity increases in both *in vivo* and *in vitro* experiments. The remarkable ability of wound healing has been verified using a mouse dorsal skin-infected wound model, demonstrating its great potential for wound treatment in clinical applications.

## Abstract 2

A calcium–copper zeolite gauze exhibits remarkable procoagulant and antibacterial ability, as well as good biocompatibility. The remarkable ability of wound healing has been verified using a mouse dorsal skin-infected wound model.

## Graphical abstract

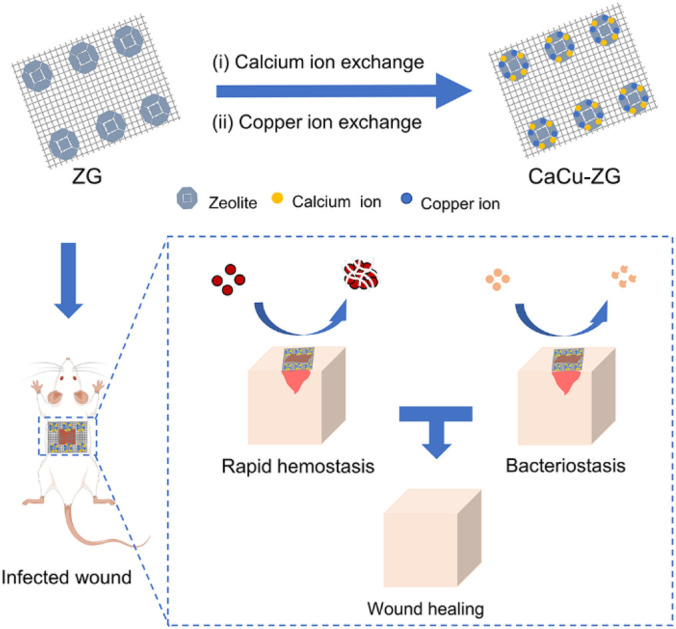

## 1. Introduction

Wound treatment is a significant global health issue.^1,2^ The wound healing process is complex and comprises four stages: hemostasis, inflammation, proliferation, and remodeling.^3,4^ Uncontrolled bleeding from trauma can significantly increase the risk of mortality.^5,6^ During the extended wound healing process, wounds are highly susceptible to bacterial infection, leading to prolonged healing time, tissue damage, and other complications.^7,8^ Wound dressings are one of the most commonly used biomaterials. However, traditional dressings like medical gauze are generally not effective in controlling wound bleeding.^9^ Instead, the medical gauze can even provide a favourable environment for bacterial growth, leading to infections.^10,11^ Therefore, there is a critical need to develop a wound dressing that can not only stop bleeding but also exhibit antibacterial property. Rapid hemostasis, excellent antibacterial activity, good biocompatibility, easy manufacturability, and low cost are essential characteristics for an ideal wound dressing.

Zeolite is a silico-aluminate material with a typical pore size of 0.4–1.2 nm, which can accommodate positively charged ions such as Ca^2+^ and Na^+^.^12,13^ The Food and Drug Administration (FDA) has approved several zeolite hemostatic products, such as QuikClot and Advanced Clotting Sponge (ACS). Its hemostatic mechanism involves absorbing water of the blood to concentrate coagulation factors and blood cells, activating the clotting cascade. Besides, Ca^2+^ released from zeolites engages in the coagulation cascade, thereby accelerating the intrinsic pathway of blood coagulation.^14–16^ Our recent studies have shown that prothrombin complexes can be assembled on the surface of calcium-based zeolites and activated into highly active thrombin.^17,18^ And we developed a zeolite–cotton composite hemostatic dressing, which has good flexibility and textile properties, effectively stopping bleeding and eliminating the risk of zeolite particles entering the human body.^19^ However, the absence of antibacterial properties in zeolite–cotton can inadvertently foster a favourable environment for bacterial growth within the blood clot, ultimately leading to the bacterial infection on the dressings. Thus, it is highly desired to develop a wound dressing that can simultaneously promote wound healing after rapidly stopping bleeding.

Inorganic antibacterial materials, such as copper, are not easily prone to resistance and are widely used. Copper exerts its antibacterial effect by releasing copper ions, which depolarize the bacterial cell membrane,^20,21^ promote the production of reactive oxygen species (ROS),^22,23^ influence enzyme activity, and induce deoxyribonucleic acid (DNA) damage.^24,25^ In recent years, there has been significant improvement in the development of novel copper-based antibacterial materials. Ren *et al.* successfully deposited copper sulfide nanoparticles onto silk fabric through *in situ* synthesis, exhibiting an impressive antibacterial rate of 99.99% against *Staphylococcus aureus* (*S. aureus*) and *Escherichia coli* (*E. coli*).^26^ Xu *et al.* introduced a novel cellulose-based Schiff base-Cu(ii) complex, which showed excellent antibacterial effects against *E. coli* and *S. aureus*.^27^ Furthermore, copper has been found to facilitate wound healing by accelerating new angiogenesis and enhancing the activity of growth factors.^28,29^

Aiming at wound treatment with efficient bleeding control and antibacterial activity, we developed a calcium–copper zeolite gauze (CaCu-ZG) by a two-step process involving calcium and copper ion exchange of zeolite gauze (ZG). The CaCu-ZG exhibits remarkable hemostatic and antibacterial properties, as well as good biocompatibility. Compared with standard medical gauze, the blood clotting time of CaCu-ZG decreased from 509 s to 282 s, and the antibacterial activity largely increased both *in vivo* and *in vitro* experiments. Notably, the copper ion exchange of ZG has no significant influence on procoagulant performance. The efficient coagulation and antibacterial properties of CaCu-ZG highlight its promising potential for wound healing.

## 2. Results and discussion

### 2.1. Preparation and characterization of CaCu-ZG

**Fig. 1:**
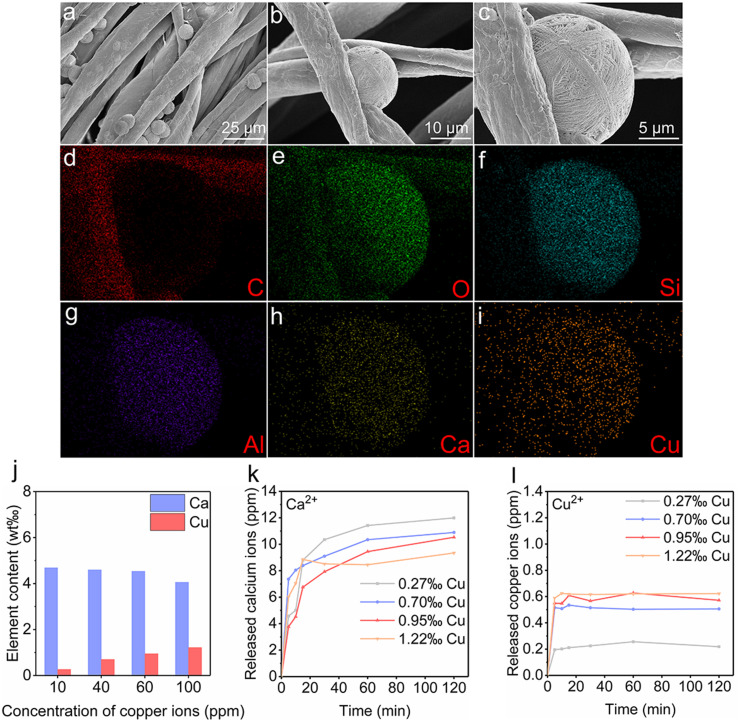
Preparation and characterization of CaCu-ZG. (a–c) SEM images of CaCu-ZG (prepared with copper ion solution at a concentration of 100 ppm); (d–i) EDS analysis of CaCu-ZG; (j) element analysis of CaCu-ZG using ICP-OES, which was prepared with copper ion solution at concentrations of 10, 40, 60, and 100 ppm; (k) released calcium ions from CaCu-ZG; (l) released copper ions from CaCu-ZG.

Zeolite gauze was synthesized *via* an *in situ* growth method to incorporate zeolite onto fibers, as in our previous research.^19,30^ We successfully prepared CaCuP-ZG by exchanging calcium and copper ions with ZG. The X-ray diffraction (XRD) pattern indicated the presence of a significant amount of zeolite P (PDF no. 44-0052) and a small amount of zeolite CHA (PDF no. 34-0137) on the CaCu-ZG (Fig. S1†). Thermogravimetric analysis (TGA) revealed that the zeolite content of CaCu-ZG was 6.0 ± 0.75% (Table S1†). Scanning electron microscopy (SEM) images of CaCu-ZG (Fig. 1a–c) showed the presence of spherical particles, approximately 15 μm in diameter, on the cotton fibers of zeolite gauze. Energy dispersive spectroscopy (EDS) analysis confirmed the successful exchange of calcium and copper ions with the zeolite (Fig. 1d–i). Inductively coupled plasma optical emission spectrometer (ICP-OES) analysis was further used to quantify the calcium and copper ion content of CaCu-ZG. CaCu-ZG samples were prepared using copper ion solution at concentrations of 10, 40, 60, and 100 ppm, and their calcium and copper content were measured. The analysis revealed that both the calcium and copper content in CaCu-ZG samples were extremely low (Fig. 1j). Based on the copper content, they were named 0.27‰ Cu, 0.70‰ Cu, 0.95‰ Cu, and 1.22‰ Cu.

To achieve rapid coagulation and effective bacteriostasis, the release of calcium and copper ion content was a critical parameter. We conducted an *in vitro* ion release experiment on CaCu-ZG samples. As shown in Fig. 1k and l, CaCu-ZG samples exhibited rapid release of calcium and copper ions in a phosphate buffered saline (PBS) solution within 5 minutes. And the equilibrium of calcium and copper ions was achieved within 15 minutes. The rapid release of calcium ions from CaCu-ZG samples can facilitate blood coagulation to ensure hemorrhage control, and the copper ions in the blood clot can eliminate bacteria proliferation around the wound site. The concentration of calcium and copper ions released by CaCu-ZG samples remained within a safe range. In the case of CaCu-ZG (1.22‰ Cu), the released calcium and copper ions were determined to be less than 12 ppm and 0.7 ppm respectively. The typical serum calcium concentration and copper concentration were 94–124 ppm and 0.9–1.6 ppm, respectively, which clearly indicates that the ions released by the CaCu-ZG sample do not pose any risk of human poisoning.^31,32^ The CaCu-ZG samples synthesized using the two-step ion exchange method can be an efficient and biocompatible material, ensuring their safety for wound treatment.

### 2.2. *In vitro* plasma/blood clotting activity of CaCu-ZG

**Fig. 2:**
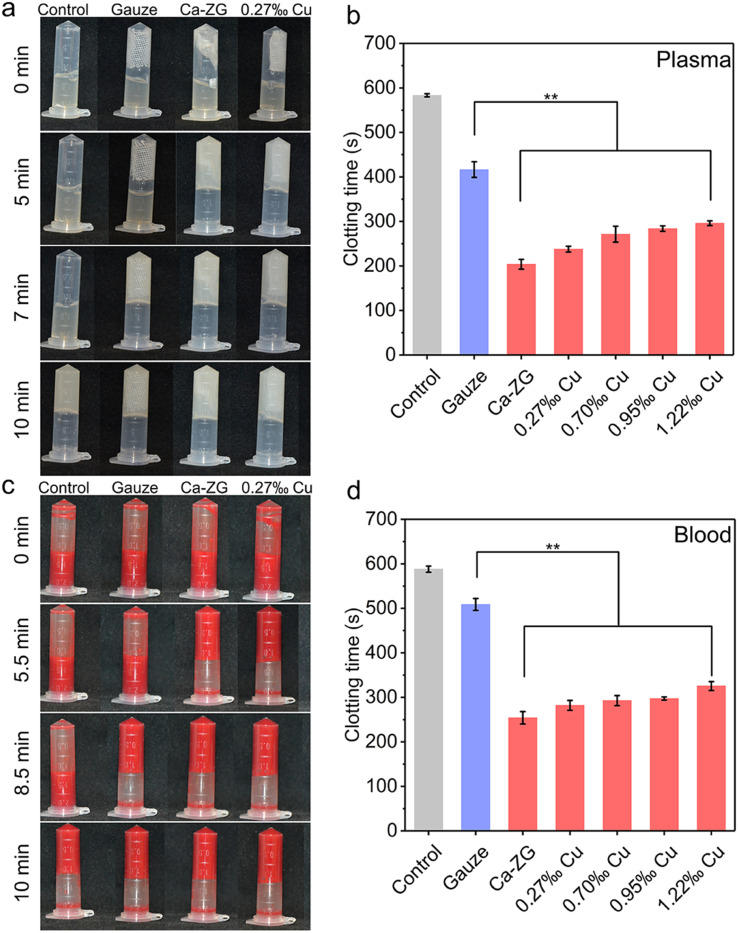
Procoagulant performance of CaCu-ZG. Photographs of blank control, gauze, calcium zeolite gauze (Ca-ZG), and CaCu-ZG in the *in vitro* (a) plasma and (c) blood clotting assay, respectively; (b and d) plasma/blood clotting time of blank control, gauze, Ca-ZG, and CaCu-ZG, respectively, **p < 0.01, *n* = 3. Error bars, mean ± SD.

The procoagulant activity was evaluated using an *in vitro* plasma/blood clotting assay. Clotting time is defined as the time required for plasma or blood to transition from a recalcified solution into a solid clot that adheres to the inner wall of a polystyrene tube. As shown in Fig. 2a, c, S2 and S3,† the clotting time of recalcified plasma and blood without interference was 583 ± 4 s and 588 ± 7 s, respectively. The clotting time of standard medical gauze for plasma and whole blood was 416 ± 18 s and 509 ± 13 s (Fig. 2b and d), respectively. CaCu-ZG samples exhibited excellent procoagulant effects in both plasma and whole blood compared to standard medical gauze. The clotting time of CaCu-ZG (0.27‰ Cu) was shortened to 238 ± 7 s (plasma) and 282 ± 11 s (blood), respectively. The specific interaction of zeolite and plasma proteins plays a critical role in procoagulant activity.^33–35^ Free copper ions in the plasma or blood could prolong clotting time (Fig. S4 and S5†). Notably, CaCu-ZG (1.22‰ Cu) still exhibited procoagulant properties (296 ± 5 s, plasma), indicating that the copper ion exchange of ZG has no significant influence on procoagulant performance.

### 2.3. *In vitro* antibacterial evaluation of CaCu-ZG

**Fig. 3:**
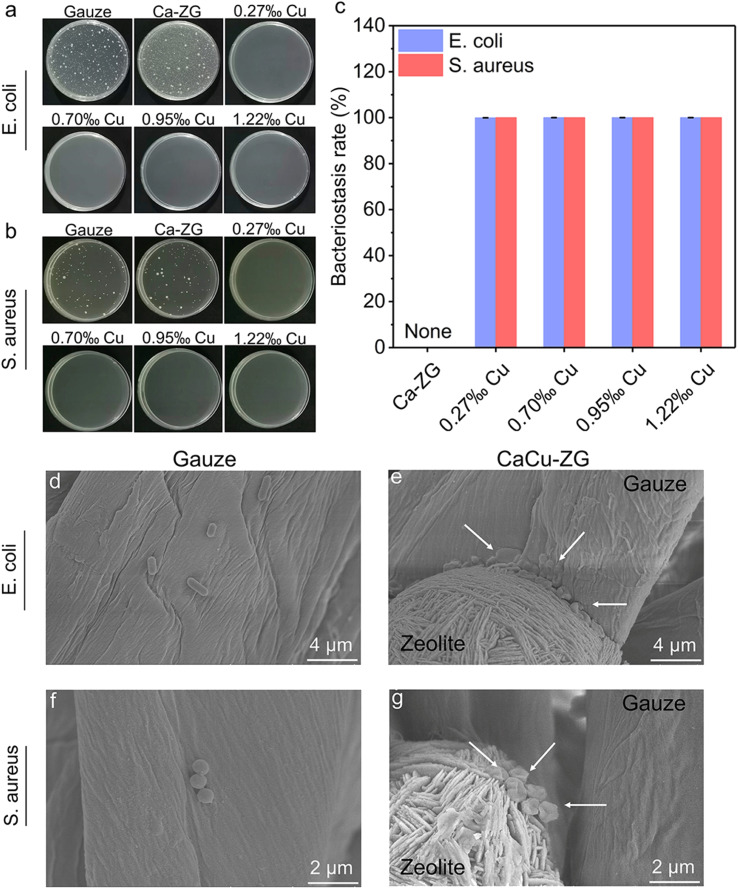
Antibacterial performance of CaCu-ZG (a) photographs of *E. coli* colonies treated with gauze, Ca-ZG, and CaCu-ZG, respectively; (b) photographs of *S. aureus* colonies treated with gauze, Ca-ZG, and CaCu-ZG, respectively; (c) bacteriostasis rate of Ca-ZG and CaCu-ZG, *n* = 3, error bars, mean ± SD; (d and e) SEM images of *E. coli* treated with gauze and CaCu-ZG (1.22‰ Cu), respectively; (f and g) SEM images of *S. aureus* treated with gauze and CaCu-ZG (1.22‰ Cu), respectively.

*In vitro* antibacterial performance was assessed using the oscillating method. As presented in Fig. 3a and b, medical gauze and Ca-ZG exhibited no obvious antibacterial activity. CaCu-ZG samples demonstrated an outstanding bacteriostatic rate of 99.9% against both *E. coli* and *S. aureus* (Fig. 3c), indicating its exceptional and broad-spectrum antibacterial efficacy. The copper ion within the zeolite of CaCu-ZG released when exposed to a bacterial suspension, which can effectively eliminate the bacteria.^36^

Furthermore, the morphology of bacteria on standard medical gauze and CaCu-ZG (1.22‰ Cu) was observed using SEM. The results revealed that the bacterial structures of *E. coli* and *S. aureus* on CaCu-ZG were visibly damaged, with the cell membrane appearing sunken or even ruptured (Fig. 3d–g). In contrast, the morphology of bacteria on the standard medical gauze was relatively intact compared to untreated bacteria (Fig. S6†). This suggests that the CaCu-ZG can disrupt the cell structure of bacteria, which will cause the bacteria to die. We believe that CaCu-ZG has high antibacterial properties, which can effectively prevent bacterial wound infections.

### 2.4. *In vitro* biocompatibility of CaCu-ZG

**Fig. 4:**
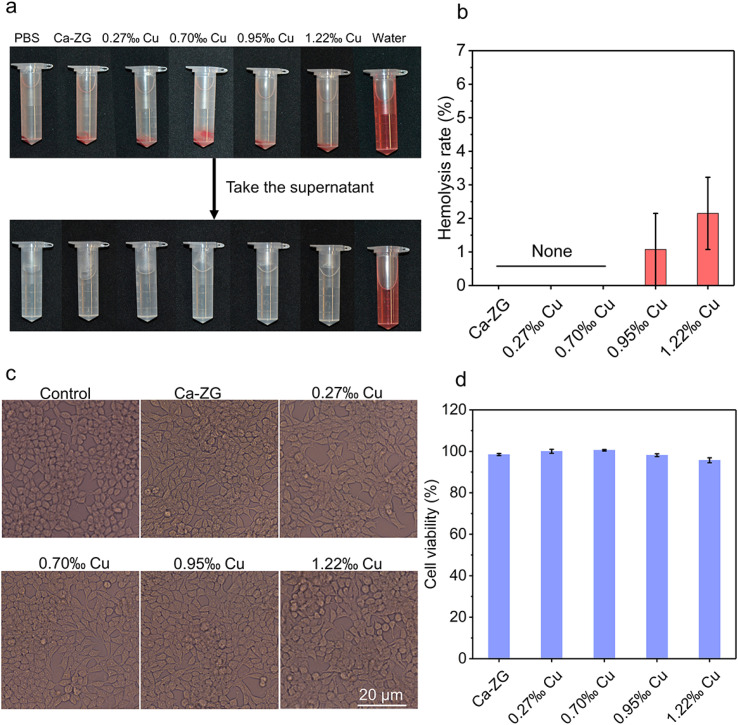
*In vitro* biocompatibility of CaCu-ZG. (a) Photographs of PBS (negative control), gauze, Ca-ZG, CaCu-ZG, and ultrapure water (positive control) in the hemolytic activity assay; (b) hemolysis rate of Ca-ZG and CaCu-ZG, *n* = 3. Error bars, mean ± SEM; (c) microscopy images of 3T3 cells treated with control, Ca-ZG and CaCu-ZG, respectively; (d) cell viability of 3T3 cells cultured with extracts of Ca-ZG and CaCu-ZG, *n* = 5. Error bars, mean ± SD.

Excellent hemocompatibility is a crucial characteristic of wound dressings. To assess the hemocompatibility of CaCu-ZG, an *in vitro* direct contact hemolytic activity experiment was conducted. Similar to the blank group (PBS), the red blood cells in suspension were almost unruptured with light yellow supernatant after being exposed to the CaCu-ZG samples at 37 °C for 1 hour (Fig. 4a). Additionally, the hemolysis ratios of all CaCu-ZG samples were assessed through supernatant absorbance detection, with ultrapure water serving as a positive control for complete red blood cell rupture. As shown in Fig. 4b, the hemolysis ratios of all CaCu-ZG samples were below 3%, indicating the outstanding hemocompatibility of CaCu-ZG.

For the application of hemostatic agents and wound dressings, the materials should exhibit favorable cytocompatibility. The cytocompatibility of as-prepared CaCu-ZG samples was evaluated using a leaching pattern method. As shown in Fig. 4c and d, none of the CaCu-ZG samples exhibited any cytotoxic leaching content. The cell morphology of the CaCu-ZG groups was similar to that of the control group, indicating a healthy condition. Then the cytocompatibility was further demonstrated through the cell survival rate, with Ca-ZG and CaCu-ZG samples surpassing a 95% relative cell survival rate (Fig. 4d). CaCu-ZG demonstrated good biocompatibility, ensuring its safe application in the treatment of patients' wounds without any biosafety concerns.

### 2.5. Stability and durability of CaCu-ZG

**Fig. 5:**
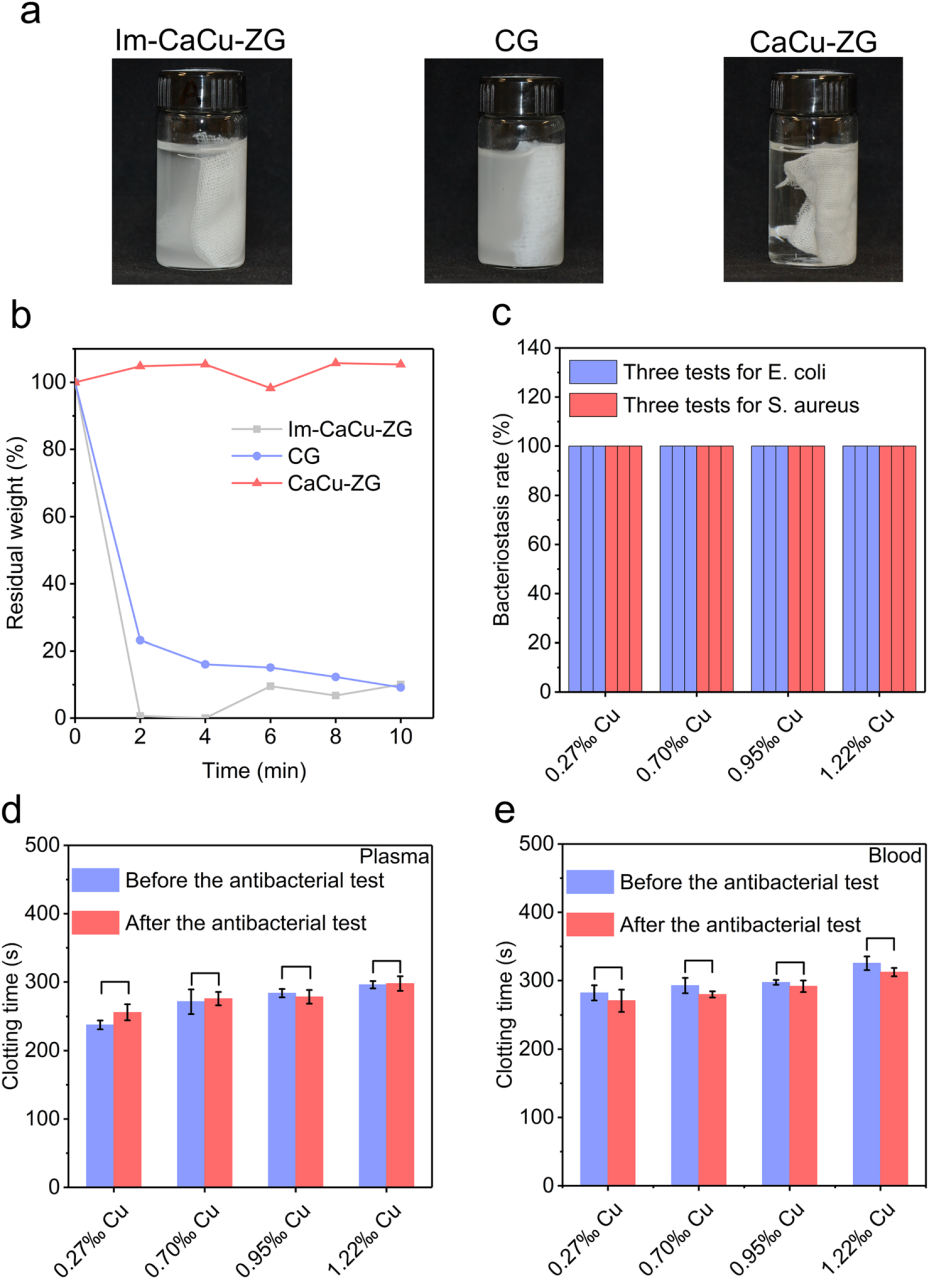
Stability and durability of CaCu-ZG. (a) Photographs of Im-CaCu-ZG, CG, and CaCu-ZG (1.22‰ Cu) after soaking into the ultrapure water; (b) relative residual weight of active ingredients on Im-CaCu-ZG, CG, and CaCu-ZG (1.22‰ Cu) after different ultrasonic times; (c) bacteriostasis rate of CaCu-ZG in three repeated bacteriostasis experiments (*n* = 3); error bars, mean ± SD. (d and e) Plasma/blood clotting time of CaCu-ZG before and after the antibacterial test (*n* = 3). Error bars, mean ± SD.

Fabric-based wound dressings are widely used in clinical practice, offering irreplaceable advantages such as easy application and excellent shape adaptivity. The stability and durability of wound dressings are critical for their clinical efficacy and safety. Among the various products available, Combat Gauze (CG, Z-Medica) impregnated with kaolin has demonstrated effectiveness in diverse situations. However, there is a potential risk associated with the leakage of kaolin into the wound. We prepared another zeolite-impregnated sample in which the calcium/copper zeolite suspension was simply dropped onto the standard medical gauze and then dried (denoted as Im-CaCu-ZG). Upon immersion of Im-CaCu-ZG and CG, a noticeable loss of kaolin or zeolite particles was observed (Fig. 5a). In contrast, the water in the CaCu-ZG (1.22‰ Cu) group remained clear. Furthermore, significant leaching was observed in the Im-CaCu-ZG and CG groups after just 2 minutes of sonication, with over 70% of kaolin or zeolite particles being released (Fig. 5b).

The antibacterial durability of CaCu-ZG was further assessed. Through three consecutive antibacterial experiments, CaCu-ZG demonstrated an impressive antibacterial rate exceeding 99.9% against both *E. coli* and *S. aureus* (Fig. 5c). Moreover, even after undergoing three antibacterial experiments, there was no significant change in the procoagulant activity of CaCu-ZG (Fig. 5d and e). This outstanding performance can be attributed to the robust binding between zeolite and gauze fiber as well as the sustained release of ion ions, ensuring the remarkable antibacterial durability of CaCu-ZG.

### 2.6. *In vivo S. aureus*-infected wound healing

**Fig. 6:**
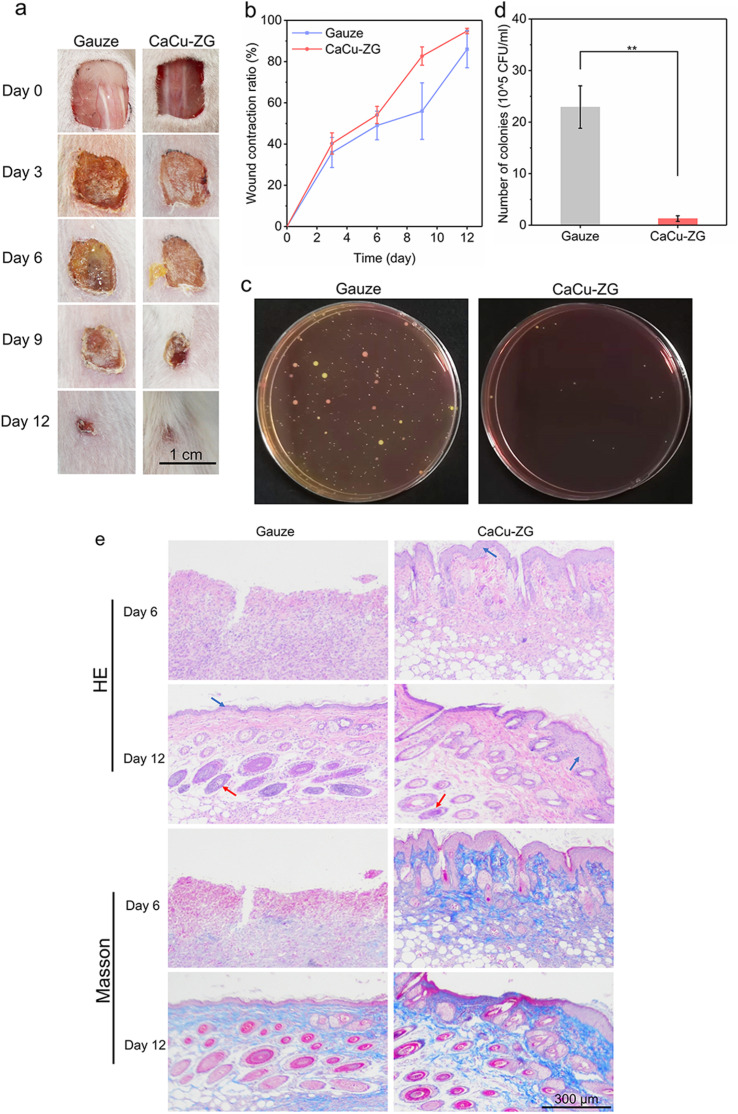
Wound healing of CaCu-ZG in a mouse back-infected wound model. (a) Photographs of wound areas after treatment with gauze and CaCu-ZG (1.22‰ Cu) on different days, respectively; (b) wound contraction ratio of mice after treatment with gauze and CaCu-ZG (1.22‰ Cu) on different days; error bars, mean ± SEM. (c) Photograph of *S. aureus* colonies sampled from the wound tissues on day 6; (d) number of colonies in the wounds of mice after treatment with gauze and CaCu-ZG (1.22‰ Cu) on day 6; **p < 0.01, *n* = 5; error bars, mean ± SD. (e) HE and Masson's trichrome staining of wound tissues on day 6 and 12 after gauze and CaCu-ZG (1.22‰ Cu) treatments. The red arrow indicates a new hair follicle, and the blue arrow indicates epithelial growth.

The *in vitro* results demonstrated that CaCu-ZG with excellent procoagulant capacity, antibacterial activity, and good biocompatibility, showed promising application in wound healing. A mouse full-thickness skin-infected wound model was applied to estimate wound closure and healing performance. Skin wounds (1 cm × 1 cm, full thickness) infected with *S. aureus* were made on the backs of mice, and the wounds were then treated with CaCu-ZG (1.22‰ Cu) and standard medical gauze. As depicted in Fig. 6a and b, after healing for 6 days, the wounds treated with medical gauze showed more obvious contamination with pathogens compared with the wounds treated with CaCu-ZG (1.22‰ Cu). To quantify the bacteria concentration, wound tissues after healing for 6 days were collected from mice, which were subsequently homogenized and incubated to observe the colonies of *S. aureus* (Fig. 6c and d). The *in vivo* antibacterial performance of CaCu-ZG (1.22‰ Cu) was assessed by examining viable bacteria colonies. The results clearly demonstrate that the concentration of bacteria in the CaCu-ZG (1.22‰ Cu) group was significantly lower (1.3 × 10^5^ CFU mL^−1^) compared to the gauze group (2.3 × 10^6^ CFU mL^−1^). It provides reliable evidence of the excellent antibacterial properties of CaCu-ZG (1.22‰ Cu) and effectively promotes wound closure in the early stages through antibacterial activity.

The wounds treated with CaCu-ZG (1.22‰ Cu) showed superior closure compared to those treated with standard medical gauze (Fig. 6a). On day 9, the wound closure percentages of the gauze group and CaCu-ZG (1.22‰ Cu) group were 56.0 ± 13.7% and 82.7 ± 4.5% (*n* = 5), respectively. On day 12, the wound contraction ratios of the gauze group and CaCu-ZG (1.22‰ Cu) group were 86.0 ± 9.0% (*n* = 5) and 95.0 ± 1.3% (*n* = 5), respectively (Fig. 6b). These results clearly demonstrate the effective wound healing promotion achieved by CaCu-ZG with procoagulant and antibacterial activity. These findings are highly significant, as bacterial infections can impede wound healing and lead to severe complications. The ability of CaCu-ZG to prevent bacterial infection highlights its potential as a promising wound dressing material.

Wound healing is a comprehensive process consisting of four overlapping phases: hemostasis, inflammation, proliferation, and wound remodeling with scar tissue formation. Hematoxylin–eosin (HE) and Masson's trichrome staining were employed to further investigate the internal details of wound healing (Fig. 6e). HE staining was used to evaluate the wound morphology, inflammatory response, and tissue growth.^37^ On day 6, the wound tissue in the medical gauze group exhibited inflammatory responses and no obvious epithelialization, whereas the wound tissue treated with CaCu-ZG (1.22‰ Cu) showed less inflammatory infiltration, partial epithelialization, and new hair follicles. On day 12, wound tissue epithelialization and hair follicles were observed, and inflammation was absent in both the medical gauze and CaCu-ZG (1.22‰ Cu) groups. More hair follicles were formed in the wound tissues of the CaCu-ZG (1.22‰ Cu) group, indicating excellent wound healing. Masson's trichrome staining was used to evaluate collagen deposition, and the blue color marked the collagen and the red color marked the keratin or muscle fiber.^38^ The results demonstrated that the wound tissue showed obvious collagen deposition after healing for 12 days in the medical gauze and CaCu-ZG (1.22‰ Cu) groups, and the collagen fibers of the skin tissue treated with CaCu-ZG (1.22‰ Cu) were denser and better arranged.

Finally, HE staining was performed on the organs of the heart, liver, spleen, lung, and kidney of mice, and the results showed no significant abnormalities in the organ structure (Fig. S7†). These results confirmed that CaCu-ZG is a biocompatible and safe wound dressing material.

## 3. Conclusion

In summary, we have successfully developed a wound dressing CaCu-ZG through a two-step process involving the exchange of calcium and copper ions in zeolite gauze. The exchange of copper ions in ZG does not significantly affect its outstanding ability to promote blood clotting. CaCu-ZG exhibits a delightful release of calcium and copper ions, ensuring significant procoagulant and antibacterial performance for wound tissue as well as favorable biocompatibility. The remarkable capacity to close and heal wounds has been verified through *in vivo* experiments using a full-thickness skin-infected wound model. With excellent procoagulant activity, efficient antibacterial activity, good biocompatibility, exceptional stability, and durability, CaCu-ZG offers an effective solution for healing infected wounds. This work provides promising potential for clinical application in wound management.

## 4. Experimental section

### 4.1. Materials

Standard medical gauze was purchased from Nanchang Grace Medical Materials Co., Ltd. Zeolite gauze was obtained from Hangzhou Zeolite Innovation Medical Technology Co., Ltd. Calcium chloride was purchased from Sinopsin Group Chemical Reagent Co., Ltd. Copper nitrate trihydrate was purchased from Shanghai Maclin Biochemical Technology Co., Ltd. Bovine plasma was obtained from Chuzhou Shinoda Biotechnology Co., Ltd. Rabbit blood was obtained from the Laboratory Animal Center of Zhejiang University. *E. coli* CMCC (B) 44102 was purchased from Shanghai Zhiqiao Bioengineering Co., Ltd. *S. aureus* (ATCC25923) was purchased from the China Medical Bacteria Preservation Management Center. Nutrition agar medium was purchased from Beijing Sanyao Technology Development Co., Ltd. Mannitol sodium chloride agar was purchased from Guangdong Huankai Microbial Technology Co., Ltd. CG (Z-Medica, USA) was purchased from a commercial source. All materials were used without further purification.

### 4.2. Preparation of CaCu-ZG

#### 4.2.1. Calcium ion exchange of ZG

To obtain Ca-ZG, zeolite gauze was soaked in a calcium chloride solution (5 M) at a ratio of 0.05 g mL^−1^. The mixture was then oscillated at room temperature for 3 h. Subsequently, the liquid was replaced with a fresh solution, and the zeolite gauze was oscillated again for another 3 h. Once the exchange process was completed, the Ca-ZG was washed and dried.

#### 4.2.2. Copper ion exchange of Ca-ZG

The experimental conditions for the copper ion exchange process were identical to those used for the calcium ion exchange process, except for the substitution of the solution with copper ion solution (pH = 3) at concentrations of 10, 40, 60, and 100 ppm. After the exchange process, the zeolite gauze was meticulously washed and dried to prepare a series of CaCu-ZG samples.

### 4.3. Preparation of Im-CaCu-ZG

The commercially obtained zeolite was subjected to ion exchange using a solid–liquid ratio of 0.2 g mL^−1^, and the experimental conditions for the ion exchange process were identical to those used for CaCu-ZG preparation, with a 5 M calcium chloride solution and a 100 ppm copper ion solution. The obtained zeolite was denoted as CaCu-Z. CaCu-Z (150 mg) was then dispersed in anhydrous ethanol to prepare a zeolite suspension, which was evenly dripped on the gauze (1 g) and dried to obtain the sample (denoted as Im-CaCu-ZG). The TGA revealed that the average loading content of CaCu-Z was about 6.8 wt%, which was similar to that of CaCu-ZG (6.0 wt%).

### 4.4. Characterizations

The surface morphology and chemical composition of the samples were observed and analyzed *via* the field-emission scanning electron microscope (FE-SEM, Hitachi SU8010, Japan). The XRD (Bruker D8 Advance) patterns of the samples were recorded on a Rigaku Ultima IV with Cu Kα radiation (10° min^−1^). The thermogravimetry analysis (METTLER, TGA/DSC 1/1100, Switzerland) was recorded under dynamic oxygen flow by heating the samples to 800 °C at a rate of 10 °C min^−1^. The element content of the samples was analyzed by ICP-OES (Thermo Fisher iCAP6300).

### 4.5. Ion content released by CaCu-ZG

The experimental procedure involved immersing the samples in PBS solution at a ratio of 0.01 g mL^−1^. The mixture was then oscillated at 37 °C for different durations. Afterward, the solution was extracted, acidified, and filtered through a 0.22 μm filter. The ion content released by the samples was analyzed by the inductively coupled plasma mass spectrometry (ICP-MS, Agilent Technologies 7800).

### 4.6. *In vitro* plasma/blood clotting assay

In order to evaluate the procoagulant activity of CaCu-ZG, we performed *in vitro* plasma and blood coagulation measurements. The clotting time is defined as the time needed to activate the coagulation cascade and cause the appearance of solid clots adhering to the wall of polystyrene. To ensure a common zero clotting time, the plasma and blood were recalcified in the experiment. The plasma and blood were recalcified to ensure a common zero coagulation time. Briefly, we weighed 15 mg of the sample into a 2 mL polystyrene tube, then added 1 mL of bovine plasma or rabbit blood and a certain amount of 0.2 M calcium chloride solution. The clotting measurement was carried out at 37 °C, and the clotting time was recorded.

### 4.7. *In vitro* antibacterial evaluation of CaCu-ZG

#### 4.7.1. *In vitro* antibacterial property

*E. coli* and *S. aureus* were selected as the bacterial strains to evaluate the antibacterial properties of CaCu-ZG. The activated *E. coli* and *S. aureus* were taken out, and the bacterial concentration was adjusted to 10^4^ CFU mL^−1^. CaCu-ZG (5 mm × 5 mm, 0.75 g) was sterilized and added to a conical bottle. 75 mL of the bacterial suspension was then added to the conical bottle. The mixture was vibrated for 18 h in a 37 °C bacteriological incubator. Afterward, the bacterial suspension was removed and diluted. 1 mL of the diluted bacterial suspension was transferred to a Petri dish, and a nutrient ager solution was poured into the dish. Once the culture liquid solidified, the Petri dish was inverted and placed in the incubator. The colonies were then counted to calculate the bacteriostasis rate. Medical gauze was used as the control group, and three samples were repeated in each group. The formula for calculating the bacteriostatic rate was as follows:
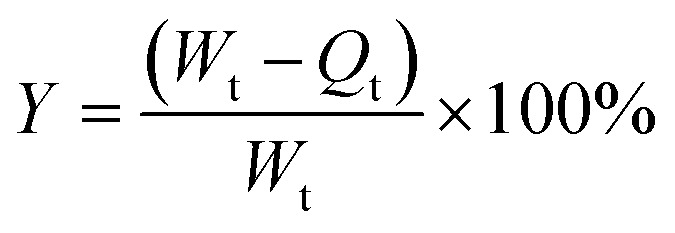
where *Y* represents the antibacterial rate, *W*_t_ is the concentration of viable bacteria in the conical flask after 18 h in the control group, and *Q*_t_ is the concentration of viable bacteria after 18 h in the experimental group.

#### 4.7.2. Effect of CaCu-ZG on bacterial morphology

The sterilized CaCu-ZG (1.22‰ Cu) was cut into 1 cm × 1 cm and placed in a 12-well plate. Then, 1 mL of bacterial suspension (1 × 10^7^ CFU mL^−1^) was added to the 12-well plate. The mixture was then incubated for 18 h at 37 °C in a bacteriological incubator. After incubation, the CaCu-ZG (1.22‰ Cu) was cleaned with PBS and fixed with glutaraldehyde (2.5 vol%) overnight at 4 °C. The sample was subjected to gradient dehydration using different concentrations of ethanol. The sample was then dried at room temperature and observed using SEM.

### 4.8. Biocompatibility of CaCu-ZG

#### 4.8.1. Blood compatibility of CaCu-ZG

The blood compatibility test was conducted following the protocols described in previously published literature.^39,40^ Fresh rabbit blood obtained from the Laboratory Animal Center of Zhejiang University was centrifuged to separate erythrocytes. The obtained erythrocytes were then washed three times with PBS and diluted to 5% (v/v). The sample was cut into 0.5 cm × 0.5 cm squares and placed into a 2 mL centrifuge tube containing 1.6 mL of PBS. Subsequently, 0.2 mL of 5% (v/v) erythrocyte suspension was added. 0.2 mL of 5% (v/v) erythrocyte suspension was added to 1.6 mL of PBS and ultrapure water, respectively, to serve as negative and positive controls. After incubation at 37 °C for 1 h and centrifugation at 1000 rpm for 10 min, the supernatant was collected, and its absorbance at 540 nm was measured using an ultraviolet spectrophotometer (Shimadzu, UV-1900i). Finally, the hemolysis rate was calculated using the following formula:
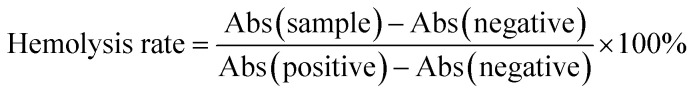
where Abs(sample) is the absorbance of the experimental group, Abs(positive) is the absorbance of the positive control group, and Abs(negative) is the absorbance of the negative control group.

#### 4.8.2. Cytotoxicity of CaCu-ZG

The procedures for evaluating the cytotoxicity of CaCu-ZG were adapted from previously published literature.^41,42^ The sample was immersed in serum-free Dulbecco's modified eagle medium (DMEM) at a ratio of 6 cm^2^ mL^−1^. After 24 h of incubation at 37 °C, the solution was filtered through a 0.22 μm filter to obtain the sample extraction. 3T3 cells were seeded in a 96-well plate at a density of 10 000 cells per well. Next, 100 μL of sample extraction was added to replace the original culture medium when the cells were attached to the wall. The negative control group was serum-free DMEM, and the positive control group was polyethylenimine (PEI) solution (1 mg mL^−1^, the molecular weight was 25 kD). Each group had 5 replicates. After 24 h, 100 μL of thiazolyl blue tetrazolium bromide (MTT) solution was added to each well to replace the sample extraction. After 2 h, 100 μL of dimethyl sulfoxide (DMSO) solution was added to dissolve the crystals. The absorbance of the solution at 570 nm was measured by a microplate reader. The relative cell viability was calculated by the following formula:
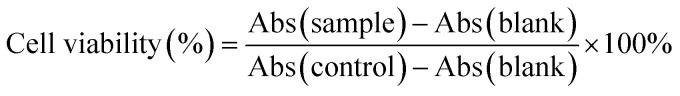
where Abs(sample) is the absorbance of the experimental group, Abs(blank) is the absorbance of the positive control group, and Abs(control) is the absorbance of the negative control group.

### 4.9. Stability and durability of CaCu-ZG

#### 4.9.1. Stability of CaCu-ZG

The sonication treatment was used to evaluate the binding strength between the zeolite and gauze with an ultrasonic cleaner (SBL-10DT, 230 W). The samples were immersed in ultrapure water and subjected to ultrasonic treatment at different time intervals. All the samples were washed with ultrapure water three times and dried for the thermogravimetric analysis.

#### 4.9.2. Durability of CaCu-ZG

The durability of antibacterial property was assessed through three repetitions of the same procedures used for *in vitro* antibacterial evaluation. Prior to each subsequent test, CaCu-ZG was meticulously washed, dried, and sterilized. Following the assessment of antibacterial durability, the procoagulant durability of the collected CaCu-ZG was evaluated using the *in vitro* plasma/blood clotting assay as mentioned above.

### 4.10. *In vivo* infected wound healing

The study adhered to the Guide for the Care and Use of Laboratory Animals. This study protocol was approved by the Laboratory Animal Ethics Committee of Zhejiang University (ZJU20220529). Female ICR mice (6–8 weeks old) were provided by the Laboratory Animal Center of Zhejiang University. 20ICR mice were randomly divided into two groups. First, mice were anesthetized by an abdominal injection of pentobarbital sodium. Full-thickness skin wounds (1 cm × 1 cm) were created on the back of each mouse. 20 μL of *S. aureus* suspension (10^8^ CFU mL^−1^) was dripped onto the back wounds. After 30 minutes, the mice of the experimental group were covered with CaCu-ZG (1.22‰ Cu) on the back wounds. The mice in the control group were covered with ordinary medical gauze. Breathable pressure-sensitive tape was used to ensure the sample was in place. The gauze samples were changed at predetermined intervals. The back wounds were photographed using a camera, and the wound area was calculated using Image-Pro Plus. Finally, the wound healing rate was calculated using the following formula:
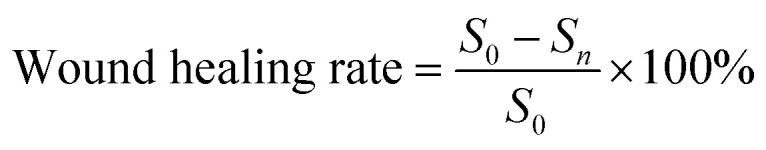
where *S*_0_ is the wound area of mice on day 0, and *S*_*n*_ is the wound area of mice on day *n*.

On day 6, five mice from each group were randomly selected and euthanized. Their wound tissues were homogenized in PBS at a ratio of 0.02 mg μL^−1^. Then the tissue homogenate was gradient-diluted. 1 mL of the diluted homogenate was added to a Petri dish and mixed with mannitol sodium chloride agar medium. After the culture liquid solidified, the Petri dish was kept in an inverted position during incubation. Finally, the colonies are counted.

On day 12, all mice were euthanized, and their hearts, liver, spleen, lungs, and kidneys were fixed with 4% paraformaldehyde and stained with H&E.

On day 6 and 12, the surrounding skin tissues of the wounds in each group were fixed with 4% paraformaldehyde and stained with H&E and Masson's trichrome.

## Author contributions

Mingtao Wang: conceptualization, methodology, investigation, formal analysis, and writing the original draft. Wenzhao Zhang: conceptualization, methodology. Chenchen Wang: data curation, investigation. Liping Xiao: conceptualization, resources, validation, writing, reviewing, and editing. Lisha Yu: conceptualization, methodology, validation, formal analysis, writing the original draft, reviewing, and editing. Jie Fan: conceptualization, resources, validation, supervision, funding acquisition, writing, reviewing, and editing.

## Conflicts of interest

There are no conflicts to declare.

## Supplementary Material

RA-014-D3RA06070E-s001

## References

[cit1] Wang Z., Rong F., Li Z., Li W., Kaur K., Wang Y. (2023). Chem. Eng. J..

[cit2] Cao X., Zhang Z., Sun L., Luo Z., Zhao Y. (2022). J. Nanobiotechnol..

[cit3] Sultana T., Hossain M., Rahaman S., Kim Y. S., Gwon J.-G., Lee B.-T. (2021). Carbohydr. Polym..

[cit4] Saghazadeh S., Rinoldi C., Schot M., Kashaf S. S., Sharifi F., Jalilian E., Nuutila K., Giatsidis G., Mostafalu P., Derakhshandeh H., Yue K., Swieszkowski W., Memic A., Tamayol A., Khademhosseini A. (2018). Adv. Drug Delivery Rev..

[cit5] Hong Y., Zhou F., Hua Y., Zhang X., Ni C., Pan D., Zhang Y., Jiang D., Yang L., Lin Q., Zou Y., Yu D., Arnot D. E., Zou X., Zhu L., Zhang S., Ouyang H. (2019). Nat. Commun..

[cit6] Eming S. A., Martin P., Tomic-Canic M. (2014). Sci. Transl. Med..

[cit7] Yao H., Wu M., Lin L., Wu Z., Bae M., Park S., Wang S., Zhang W., Gao J., Wang D., Piao Y. (2022). Mater. Today Bio.

[cit8] Wang A., Fan G., Qi H., Li H., Pang C., Zhu Z., Ji S., Liang H., Jiang B.-P., Shen X.-C. (2022). Biomaterials.

[cit9] Yu L., Zhang H., Xiao L., Fan J., Li T. (2022). ACS Appl. Mater. Interfaces.

[cit10] Shi L., Liu X., Wang W., Jiang L., Wang S. (2019). Adv. Mater..

[cit11] Li S., Chen A., Chen Y., Yang Y., Zhang Q., Luo S., Ye M., Zhou Y., An Y., Huang W., Xuan T., Pan Y., Xuan X., He H., Wu J. (2020). Chem. Eng. J..

[cit12] Guo B., Dong R., Liang Y., Li M. (2021). Nat. Rev. Chem..

[cit13] Li X., Lu P., Jia H.-R., Li G., Zhu B., Wang X., Wu F.-G. (2023). Coord. Chem. Rev..

[cit14] Pourshahrestani S., Zeimaran E., Djordjevic I., Kadri N. A., Towler M. R. (2016). Mater. Sci. Eng., C.

[cit15] Hickman D. A., Pawlowski C. L., Sekhon U. D. S., Marks J., Gupta A. S. (2018). Adv. Mater..

[cit16] Li J., Cao W., Lv X.-x., Jiang L., Li Y.-j., Li W.-z., Chen S.-z., Li X.-y. (2013). Acta Pharmacol. Sin..

[cit17] Li Y., Liao X., Zhang X., Ma G., Zuo S., Xiao L., Stucky G. D., Wang Z., Chen X., Shang X., Fan J. (2014). Nano Res..

[cit18] Shang X., Chen H., Castagnola V., Liu K., Boselli L., Petseva V., Yu L., Xiao L., He M., Wang F., Dawson K. A., Fan J. (2021). Nat. Catal..

[cit19] Yu L., Shang X., Chen H., Xiao L., Zhu Y., Fan J. (2019). Nat. Commun..

[cit20] Mitra D., Kang E.-T., Neoh K. G. (2019). ACS Appl. Mater. Interfaces.

[cit21] Shuai C., Liu L., Zhao M., Feng P., Yang Y., Guo W., Gao C., Yuan F. (2018). J. Mater. Sci. Technol..

[cit22] Applerot G., Lellouche J., Lipovsky A., Nitzan Y., Lubart R., Gedanken A., Banin E. (2012). Small.

[cit23] Li M., Ma Z., Zhu Y., Xia H., Yao M., Chu X., Wang X., Yang K., Yang M., Zhang Y., Mao C. (2016). Adv. Healthcare Mater..

[cit24] Wang Z., Liang K., Chan S.-W., Tang Y. (2019). J. Hazard. Mater..

[cit25] Lv Q., Zhang B., Xing X., Zhao Y., Cai R., Wang W., Gu Q. (2018). J. Hazard. Mater..

[cit26] Ren Y., Yan B., Wang P., Yu Y., Cui L., Zhou M., Wang Q. (2022). ACS Sustain. Chem. Eng..

[cit27] Xu Y., Shi Y., Lei F., Dai L. (2020). Carbohydr. Polym..

[cit28] Wang X., Cheng F., Liu J., Smått J.-H., Gepperth D., Lastusaari M., Xu C., Hupa L. (2016). Acta Biomater..

[cit29] Ermini M. L., Voliani V. (2021). ACS Nano.

[cit30] Zhang W., Wu J., Yu L., Chen H., Li D., Shi C., Xiao L., Fan J. (2021). ACS Appl. Mater. Interfaces.

[cit31] Lokre R. J. (1949). Indian Med. Gaz..

[cit32] Olusi S., Al-Awadhi A., Abiaka C., Abraham M., George S. (2003). Biol. Trace Elem. Res..

[cit33] González-García C., Cantini M., Ballester-Beltrán J., Altankov G., Salmerón-Sánchez M. (2018). Acta Biomater..

[cit34] Ozboyaci M., Kokh D. B., Corni S., Wade R. C. (2016). Q. Rev. Biophys..

[cit35] Marruecos D. F., Schwartz D. K., Kaar J. L. (2018). Curr. Opin. Colloid Interface Sci..

[cit36] Gross T. M., Lahiri J., Golas A., Luo J., Verrier F., Kurzejewski J. L., Baker D. E., Wang J., Novak P. F., Snyder M. J. (2019). Nat. Commun..

[cit37] Ma W., Dong W., Zhao S., Du T., Wang Y., Yao J., Liu Z., Sun D., Zhang M. (2022). Mater. Sci. Eng., C.

[cit38] Xue Y., Guo Y., Yu M., Wang M., Ma P. X., Lei B. (2017). Adv. Healthcare Mater..

[cit39] Xie H., Chen X., Shen X., He Y., Chen W., Luo Q., Ge W., Yuan W., Tang X., Hou D., Jiang D., Wang Q., Liu Y., Liu Q., Li K. (2018). Int. J. Biol. Macromol..

[cit40] Yang X., Shi N., Liu J., Cheng Q., Li G., Lyu J., Ma F., Zhang X. (2023). Adv. Healthcare Mater..

[cit41] Panawes S., Ekabutr P., Niamlang P., Pavasant P., Chuysinuan P., Supaphol P. (2017). J. Drug Delivery Sci. Technol..

[cit42] He H., Sun C., Weng Y., Huang H., Ni P., Fang Y., Xu R., Wang Z., Liu H. (2022). Carbohydr. Polym..

