# Supplementary material for: Hemostatic and antibacterial calcium–copper zeolite gauze for infected wound healing
Source: RSC Adv. 2024 Jan 2;14(2):878–88. doi: 10.1039/d3ra06070e (PMC10759188; doi:10.1039/d3ra06070e)
Supplement: RA-014-D3RA06070E-s001 [file RA-014-D3RA06070E-s001.pdf]

Electronic Supplementary Material (ESI) for RSC Advances.
This journal is © The Royal Society of Chemistry 2023

SUPPLEMENTARY INFORMATION

Hemostatic and Antibacterial Calcium-Copper Zeolite Gauze for Infected Wound Healing

Mingtao Wang,[a] Wenzhao Zhang,[a] Chenchen Wang,[a] Liping Xiao,*[,a] Lisha Yu*[,a,b] and Jie Fan*[,a]

Please do not adjust margins

Supplementary Figures

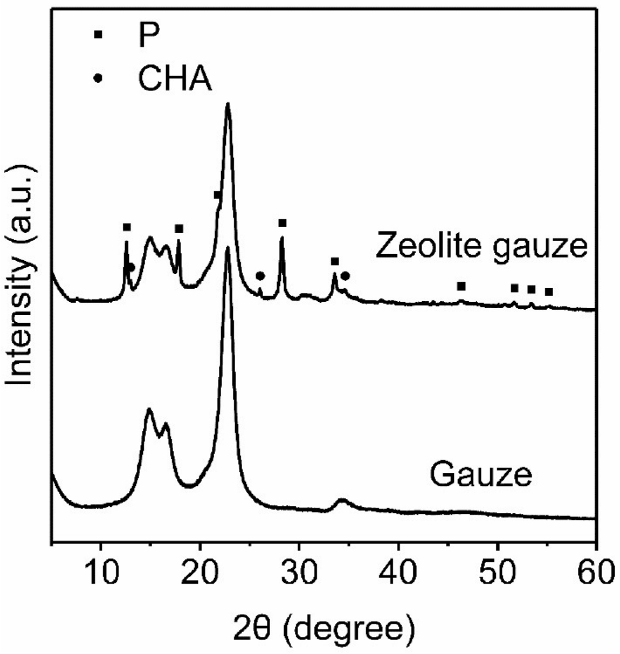

Fig. S1 XRD patterns of zeolite gauze and gauze.

 This journal is © The Royal Society of Chemistry 20xx

Please do not adjust margins

Please do not adjust margins

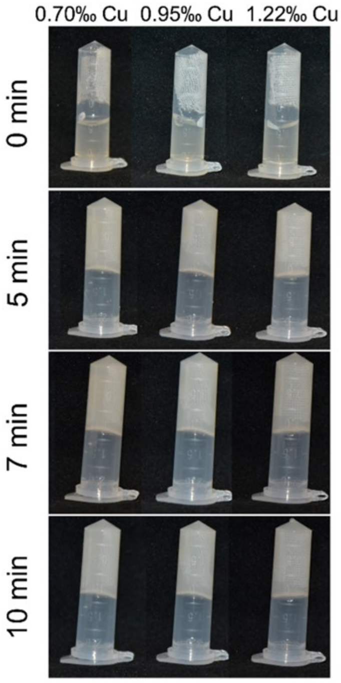

Fig. S2 Photograph of CaCu-ZG in the in vitro plasma clotting assay.

Please do not adjust margins

Please do not adjust margins

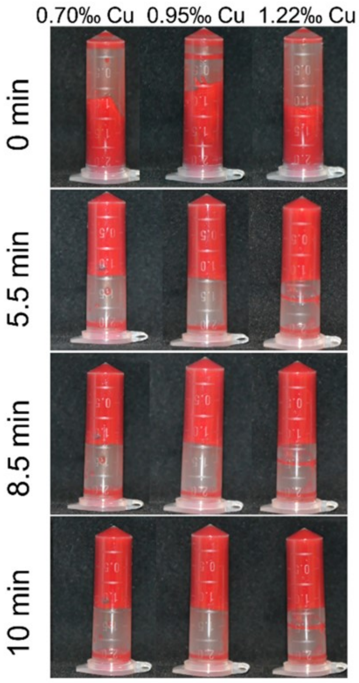

Fig. S3 Photograph of CaCu-ZG in the in vitro blood clotting assay.

This journal is © The Royal Society of Chemistry 20xx

Please do not adjust margins

Please do not adjust margins

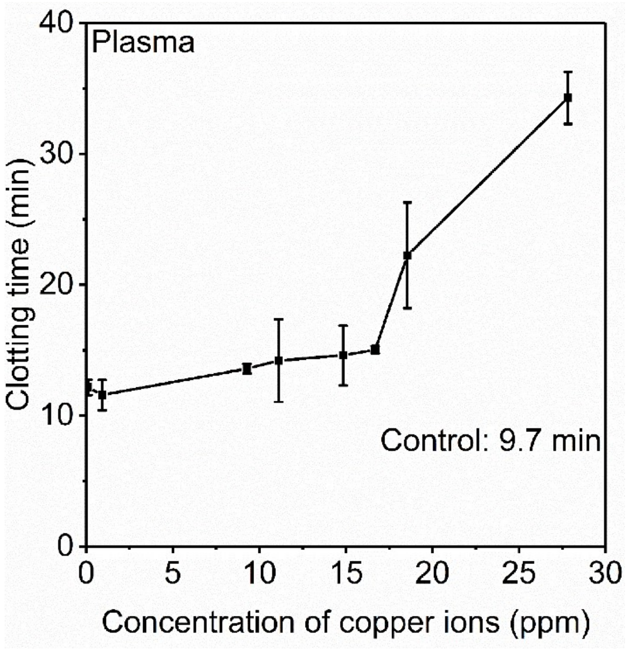

Fig. S4 Plasma clotting time of different copper ion concentrations (n = 3). Error bars, mean ± SD. The clotting time of normal plasma in the blank control group is 9.7 min.

Please do not adjust margins

Please do not adjust margins

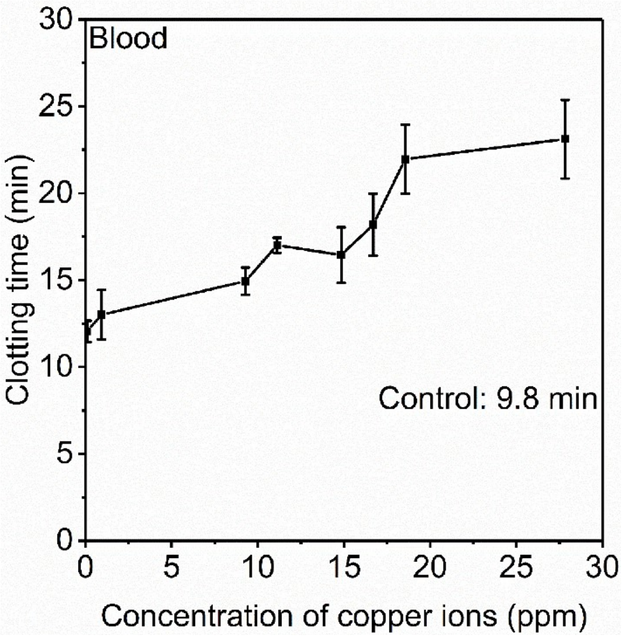

Fig. S5 Blood clotting time of different copper ion concentrations (n = 3). Error bars, mean ± SD. The clotting time of normal plasma in the blank control group is 9.8 min.

This journal is © The Royal Society of Chemistry 20xx

Please do not adjust margins

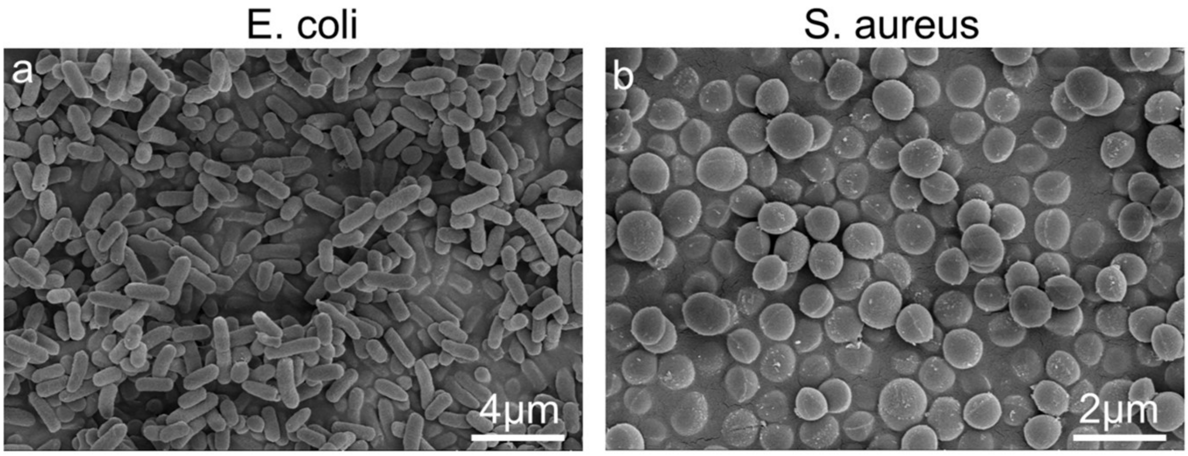

Fig. S6 (a) SEM image of E. coli without any treatment. (b) SEM image of S. aureus without any treatment.

This journal is © The Royal Society of Chemistry 20xx

Please do not adjust margins

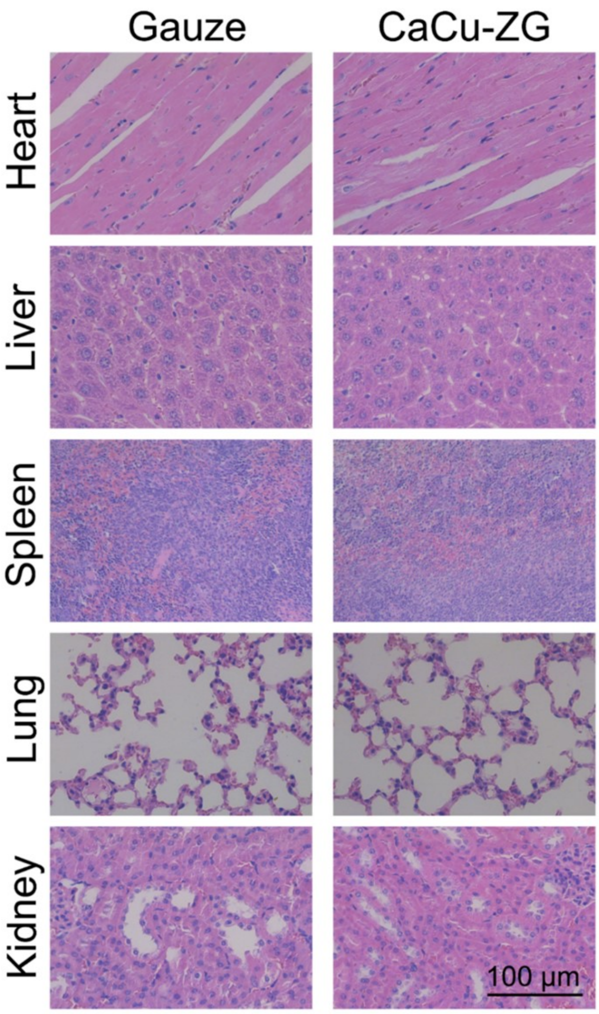

Fig. S7 HE staining for major organs of mice treated with gauze and CaCu-ZG (1.22‰ Cu).

 This journal is © The Royal Society of Chemistry 20xx

Please do not adjust margins

Please do not adjust margins

Supplementary Tables

Table S1 Content of zeolite on the surface of CaCu-ZG (prepared with copper ion solution at a concentration of 100 ppm).

Sample	1	2	3	Mean	Standard deviation
**Zeolite (%)**	5.6	6.8	5.5	6	0.75

This journal is © The Royal Society of Chemistry 20xx

Please do not adjust margins
